# Serum of patients with acute myocardial infarction prevents inflammation in iPSC-cardiomyocytes

**DOI:** 10.1038/s41598-019-42079-z

**Published:** 2019-04-04

**Authors:** Katherine Sattler, Ibrahim El-Battrawy, Zhihan Zhao, Christoph Schrottenberg, Gökhan Yücel, Huan Lan, Xin Li, Siegfried Lang, Wolfram-Hubertus Zimmermann, Lukas Cyganek, Jochen Utikal, Thomas Wieland, Ursula Ravens, Karen Bieback, Martin Borggrefe, Xiaobo Zhou, Ibrahim Akin

**Affiliations:** 1 First Department of Medicine, Faculty of Medicine, University Medical Centre Mannheim (UMM), University of Heidelberg, Mannheim, Germany; 2 DZHK (German Center for Cardiovascular Research), Partner Site Heidelberg-Mannheim and Göttingen, Göttingen, Germany; 3 Institute of Pharmacology and Toxicology, University of Göttingen, Göttingen, Germany; 4 Stem Cell Unit, Clinic for Cardiology and Pneumology, University Medical Center Göttingen, Göttingen, Germany; 5 Skin Cancer Unit, German Cancer Research Center (DKFZ), Heidelberg and Department of Dermatology, Venereology and Allergology, University Medical Center Mannheim, University of Heidelberg, Mannheim, Germany; 6 Institute of Experimental and Clinical Pharmacology and Toxicology, Medical Faculty Mannheim, University of Heidelberg, Mannheim, Germany; 7 Institue of Experimental Cardiovascular Medicine, University Heart Centre Freiburg, Bad Krozingen, Freiburg, Germany; 8 Institute for Transfusion Medicine and Immunology, Mannheim, Germany; 9 Key Laboratory of Medical Electrophysiology of the Ministry of Education, Collaborative Innovation Center for Prevention and Treatment of Cardiovascular Disease, Institute of Cardiovascular Research, Southwest Medical University, Luzhou, Sichuan China

## Abstract

Acute myocardial infarction (MI) evokes a systemic inflammatory response and locally the degradation of the necrotic tissue, followed by scar formation. The mechanisms for containment of the infarct zone are not studied well. The study aimed to examine the response of healthy cardiomyocytes to serum of patients with myocardial infarction. Human iPSC-cardiomyocytes (iPSC-CM) generated from two healthy donors were incubated with serum of patients with MI with and without ventricular fibrillation (VF) or of healthy controls. Different cell adhesion molecules were studied by flow cytometry and immunostaining. Cellular electrophysiology was studied by patch clamp. The cell adhesion molecules CD54/ICAM-1, CD58/LFA-3 and CD321/JAM-A were expressed on iPSC-CM within the plasma membrane. Incubation with serum of MI patients reduced the levels of expression of CD54/ICAM-1 and CD321/JAM-A by 15–20%. VF serum was less effective than serum of MI patients without VF. MI serum or VF serum did not affect resting potential, action potential duration or maximum depolarization velocity. Myocardial infarction serum exerts anti-inflammatory effects on healthy cardiomyocytes without affecting their electrical activity, thus helping to contain the infarct zone and to protect healthy tissue. Ventricular fibrillation during MI drives healthy cardiomyocytes towards a pro-inflammatory phenotype.

## Introduction

Myocardial infarction (MI) is a primarily local event which leads to the activation of an acute systemic inflammatory response. This is mirrored by a systemic increase of acute phase proteins, pro-inflammatory mediators, recruitment of inflammatory cells towards the myocardium and stem cell mobilization^1,2^, which ultimately initiates the stabilization of the infarcted area^3^. One of the first steps of this process is the adhesion of myeloid cells and macrophages to the infarct area to clear away the necrotic tissue^4^, followed by initiation of scar formation^3^.

For inflammatory response, distant organs producing inflammatory cells such as bone marrow or spleen are “activated” by myocardial infarction^5^. However, the systemic response can spread the inflammation to organs that are not primarily involved in the inflammatory defense such as carotid and aortic tissue, as demonstrated recently^2^. Transfer of inflammation to different organs is carried out by cytokines, a mechanism demonstrated for other disease conditions as well^6^. Although there is extensive knowledge of the inflammatory systemic and local responses after myocardial infarction, the mechanisms for containment of the infarct zone are not clear yet^3^. Therefore, in the current study we examined the response of healthy cardiomyocytes to serum of patients with myocardial infarction. Due to the limited availability of human adult ventricular cardiomyocytes, we chose induced pluripotent stem cell (iPSC)-derived cardiomyocytes of healthy human donors. These cells show proper inflammatory response upon pro-inflammatory stimuli, as demonstrated by our group recently^7^.

## Results

### Patients’ characteristics

In the current study, effects of serum of eight patients presenting with acute myocardial infarction were compared to the effects of serum of three healthy volunteers. The demographic and clinical characteristics of the patient group are shown in Tables 1 and 2. Due to the acuteness of the disease, blood drawings were undertaken after restoration of blood flow by primary percutaneous coronary intervention, thus, at the time of blood collection all patients had received acetylsalicylate and weight-adjusted heparin, as well as a second platelet aggregation inhibitor.

**Table 1:** Demographic and clinical characteristics of the patient group.

Age [years]	62 (38–75)
Male	7 (87.5)
**Clinical course**	
Cardiopulmonary resuscitation	4 (50.0)
Ventricular fibrillation requiring defibrillation	3 (37.5)
Cardiogenic shock	2 (25.0)
Impella	2 (25.0)
ECMO	2 (25.0)
1-vessel disease	0 (0.0)
2-vessel disease	1 (12.5)
3-vessel disease	7 (87.5)
Status post CABG	1 (12.5)
**Primary PCI, target vessel**	
LAD	6 (75.0)
RCX	4 (50.0)
RCA	3 (37.5)
Bypass	1 (12.5)
Number of implanted stents	3 (1–7)
abciximab	2 (25.0)
**Blood chemistry**	
Creatinine [mg/dl]	1.17 (0.5–3.4)
Creatinephosphokinase [U/l]	987.0 (116.0–2816.0)
Troponin I [µg/l]	9.53 (0.5–144.9)
Cholesterol [mg/dl]	180.5 (75.0–206.0)
Triglycerides [mg/dl]	155.0 (77.0–262.0)
Leucocyte count [10E9/l]	12.5 (4.1–23.6)
C-reactive protein [mg/dl]	5.8 (2.9–26.0)
**Medication prior to event**	
Betablockers	3 (37.5)
ACE-inhibitors/ARBs	2 (25.0)
Calcium antagonist	1 (12.5)
CSE-inhibitors	2 (25.0)
Acetylsalicylate	1 (12.5)

Continuous variables are shown as median (min-max). Quantitative variables are shown as number [%]. In several patients, more than 1 vessel was treated during PCI. Information about medication prior to event is available for 6 of 8 patients. Creatinphosphokinase levels are based on information from 7 of 8 patients.

ACE, angiotensin converting enzyme; ARB, aldosterone receptor blocker; CABG, coronary artery bypass graft; CSE, cholesterol synthesis enzyme; ECMO, extracorporeal membrane oxygenation; LAD, left anterior descending; NSTEMI, non-ST-segment elevation myocardial infarction; PCI, percutaneous coronary intervention; RCA, right coronary artery; RCX, ramus circumflexus; STEMI, ST-segment elevation myocardial infarction.

**Table 2:** Case-wise presentation of patients’ characteristics.

	Patient 1	Patient 2	Patient 3	Patient 4	Patient 5	Patient 6	Patient 7	Patient 8
Age	67	76	39	65	70	49	51	61
Sex	Male	Male	Male	Female	Male	Male	Male	Male
Immediate diagnosis	STEMI	NSTEMI	STEMI	STEMI	NSTEMI	STEMI	STEMI	STEMI
Ventricular fibrillation	Yes	Yes	Yes	Yes	No	No	No	No
Troponin I [µg/l]	5.48	5.64	144.9	1.3	0.5	13.4	76.3	87.2
CK [U/l]	455	na	2321	182	116	987	945	2816
CPR during hospital stay	Yes	Yes	Yes	Yes	No	No	No	No
Diabetes mellitus type II	Yes	Yes	No	No	Yes	No	No	No
Arterial hypertension	Yes	Yes	Yes	Yes	Yes	Yes	No	Yes
CAD	No	Yes	No	No	Yes	Yes	Yes	Yes

CAD, previously known coronary artery disease; CK, Creatinphosphokinase; CPR, cardiopulmonary resuscitation; NSTEMI, non-ST-segment-elevation myocardial infarction; STEMI, ST-segment-elevation myocardial infarction.

### Serum of patients with myocardial infarction is pro-inflammatory

Interleukin-6 (Il-6) was measured in seven serum samples of the patient group (due to lack of material of one patient) and in the serum samples of all three healthy controls. As expected, serum of patients contained higher levels of Il-6 (median 50.71 pg/ml, range 12.28–1339.65 pg/ml) than serum of healthy controls (median 4.38 pg/ml, range 2.08–16.15 pg/ml, p = 0.03).

### Expression of cell adhesion molecules on human iPSC-cardiomyocytes

**Figure 1:**
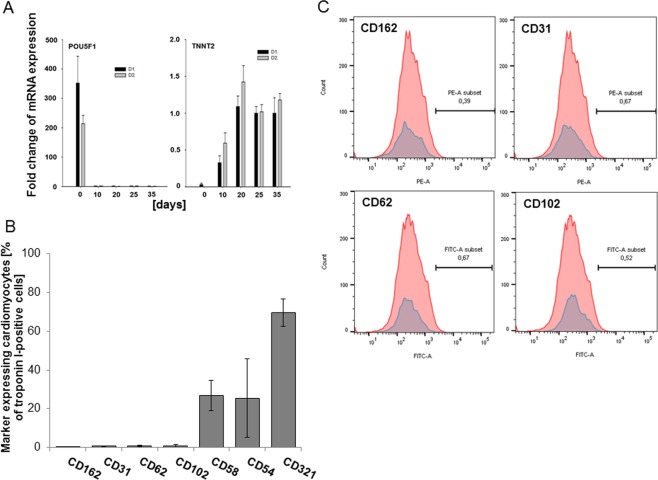
(**A**). mRNA expression of the pluripotency gene POU5F1 and of the cardiac marker troponin I (TNNT2) during differentiation of hiPSC into cardiomyocytes. The fold change over time relative to the expression of the housekeeping gene GAPDH is presented. Mean of 3 technical replicates of 3–6 biological replicates of 3 different differentiation rounds is shown. Whiskers display standard error. (**B**). Percentage of marker-expressing iPSC-cardiomyocytes (% of TNNT2-positive cells) at day 25 of differentiation. Mean of n = 5–12 measurements per marker. Whiskers display standard deviation. (**C**). CD162, CD31, CD62, and CD102 (blue) were not found on iPSC-cardiomyocytes.

For monitoring the differentiation process of hiPSC to cardiomyocytes, we performed quantitative real-time PCR of the mRNA expression of the pluripotency gene POU5F1 and the cardiomyocyte marker troponin T (TNNT2). Indeed, while the change of the expression of POU5F1 was decreased tremendously to a minimal amount at day 20 of differentiation relative to the house keeping gene GAPDH, the expression of mRNA of TNNT2 increased during the first 20 days of differentiation (Fig. 1A).

**Figure 2:**
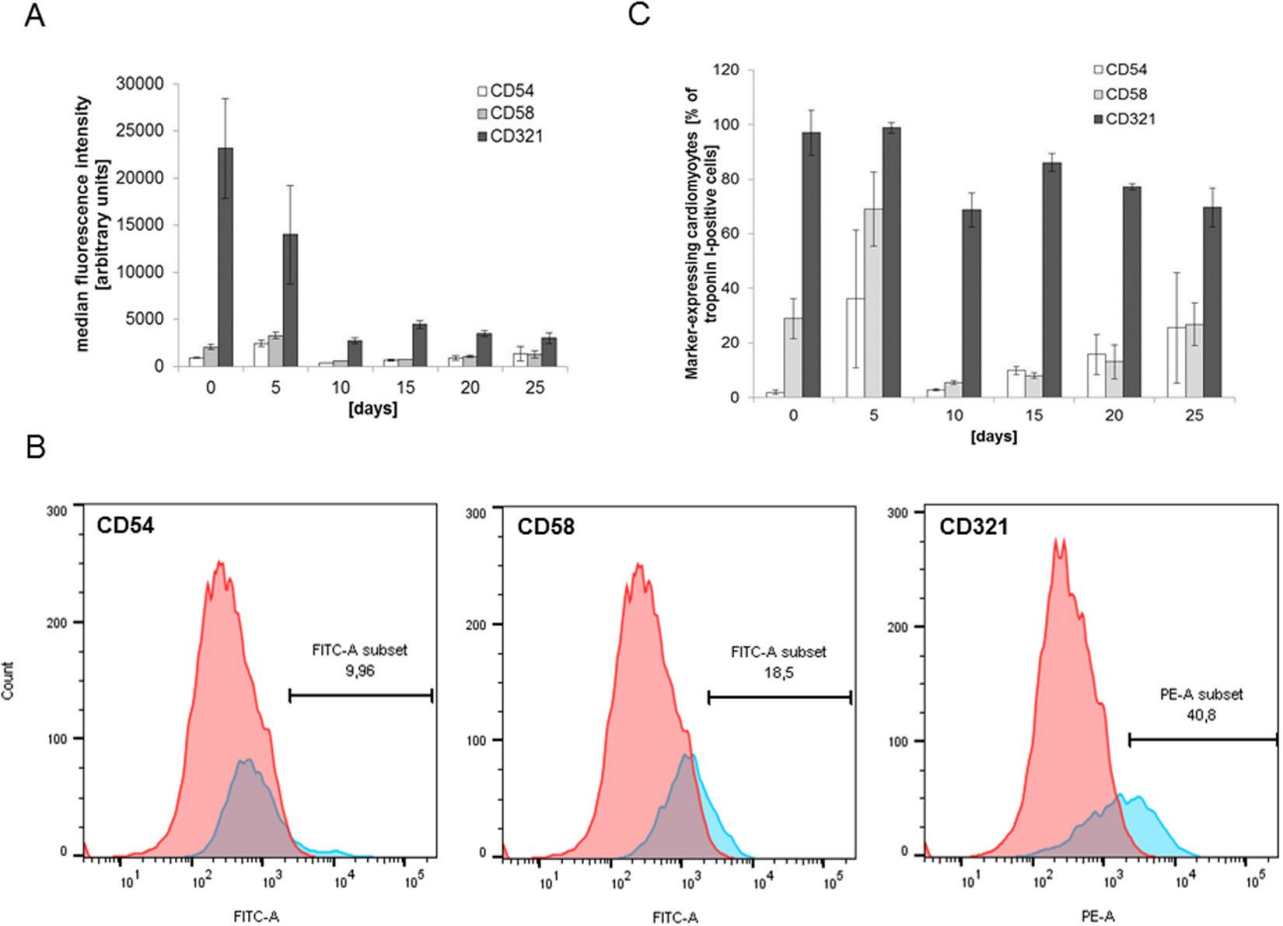
(**A**). Expression levels (median fluorescence intensity) of CD58/LFA-3, CD54/ICAM-1 and CD321/JAM-A on iPSC-cardiomyocytes (TNNT2-positive cells) at different times of differentiation. N = 5–10 measurements per marker. The mean ± standard deviation is shown. (**B**). Representative plots of flow cytometry for CD58/LFA-3, CD54/ICAM-1 and CD321/JAM-A (blue) showing the marker-positive sub-populations at differentiation day 25. The number of each marker subset gives the percentage of positive cells of all cells. (**C**). Percentage of iPSC-cardiomyocytes (% of TNNT2-positive cells) expressing CD58/LFA-3, CD54/ICAM-1 and CD321/JAM-A at different times of differentiation. N = 5–10 measurements per marker. The mean ± standard deviation is shown.

**Figure 3:**
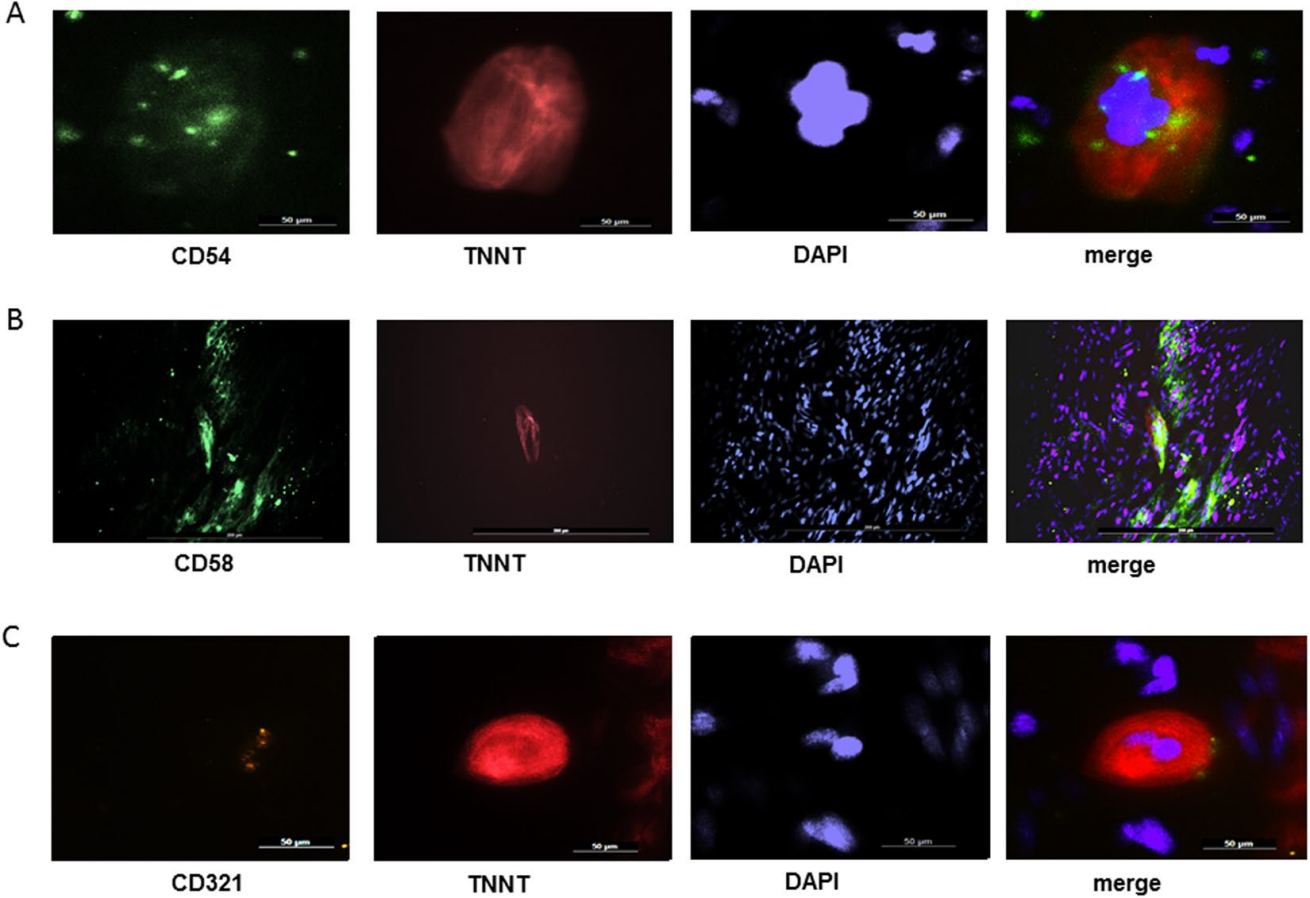
Immunofluorescence staining demonstrates the localization of CD58/LFA-3 (**A**, green), CD54/ICAM-1 (**B**, green) and CD321/JAM-A (**C**, yellow) in iPSC-cardiomyocytes (**A–C**: troponin T, red; nuclei, blue). Each marker is also expressed by cells other than cardiomyocytes, which can best be seen with CD58. Images were taken at 40x magnification. Scale bars represent 50 µm with the exception of CD58 (200 µm for better overview of marker expression by different cells).

We then tested whether hiPSC-cardiomyocytes expressed adhesion molecules during their differentiation process. At day 25, CD 31, CD 162, CD 62 and CD 102 were expressed by <2% of the cells and were thus defined as “negative” (Fig. 1B,C), while the expression of CD54/ICAM-1, CD58/LFA-3 and CD321/JAM-A was found on >2% of hiPSC-cardiomyocytes and was thus termed positive (Fig. 1B). For the three molecules CD54/ICAM-1, CD58/LFA-3 and CD321/JAM-A, a dynamic expression over time was found with stable expression at day 25 (Fig. 2A,B). At day 25, the percentage of cardiomyocyte positive for the adhesion molecules relative to all cardiomyocytes (as defined by positive expression of troponin I [TNNT2]) was 25.5%, 26.8% and 69.6% for CD54/ICAM-1, CD58/LFA-3 and CD321/JAM-A, respectively (Fig. 2C). Immunofluorescence showed the expression of all three adhesion molecules in iPSC-cardiomyocytes as well. Apart from cardiomyocytes, other cells of the cell culture expressed the markers as well as is demonstrated in the staining of CD58, leading to the impression of heavy background artefacts. However, localization in cardiomyocytes was demonstrated by positive doublestaining for the cardio-specific marker troponin I (TNNT2, Fig. 3).

### Serum of myocardial infarction alters number of stimulated cardiomyocytes

Stimulation with serum reduced the percentage of cardiomyocytes expressing the respective cell adhesion molecule when compared to unstimulated cells. However, different changes were observed regarding effect of myocardial infarction vs. control serum.

Serum of patients with myocardial infarction (MI) exerted the strongest effect on cells expressing CD58/LFA-3. When compared to the incubation with control serum, 5% (serum/medium, v/v) MI serum reduced positive cells by 40% (p = 0.02), and incubation with 20% MI serum reduced cells by 49% (p < 0.01). For CD54/ICAM-1, an increased number of cells was observed when stimulation was performed with 20% MI serum (p < 0.01 vs. control serum), while 5% MI serum did not change cell numbers. For CD321/JAM-A, cell number did not change upon incubation with MI serum compared to control serum. Table 3 shows the results in detail.

**Table 3:** Percentage of cardiomyocytes positive for cell adhesion molecules.

5% serum	controls	MI	p value
CD58/LFA-3	14.1 (11.7–16.5)	8.5 (2.6–19.2)	0.02
CD54/ICAM-1	8.1 (4.0–11.8)	5.8 (4.2–6.6)	>0.05
CD321/JAM-A	44.4 (38.6–50.0)	45.2 (35.5–66.6)	>0.05
**20% serum**	**controls**	**MI**	**p value**
CD58/LFA-3	10.2 (6.0–13.9)	5.2 (0.4–15.5)	<0.01
CD54/ICAM-1	5.4 (4.5–5.7)	7.3 (4.8–24.5)	<0.01
CD321/JAM-A	45.1 (36.5–58.9)	39.8 (33.1–100.0)	>0.05

Data are shown as median (minimum–maximum). Numbers present the percentage of cells positive for the respective marker relative to the number of all cardiomyocytes, as defined by positive TNNT2 expression. Results are based on n = 13–28 measurements per marker.

### Serum of myocardial infarction affects levels of cell adhesion molecule expression

In addition to the effects on cell numbers, incubation of cardiomyocytes with serum of patients with myocardial infarction (MI) for 48 hours resulted in changes of the expression levels of the different cell adhesion molecules, as expressed by the median fluorescence intensity (MIF) of each marker. Expression of CD58/LFA-3 was significantly reduced by approximately 15% by incubation with MI serum, irrespective of serum concentration. 5% MI serum reduced the levels of CD54/ICAM-1 by 20%, while 20% MI serum reduced the levels of CD321/JAM-A to 85%. Table 4 gives the exact data of the values of the median fluorescence intensity of each group.

**Table 4:** Median of the fluorescence intensity of cell adhesion molecules.

5% serum	controls	MI	p value
CD58/LFA-3	1272.5 (1210.0–1368.0)	1111.0 (889.0–1368.0)	0.03
CD54/ICAM-1	577.0 (459.0–927.0)	466.0 (388.0–530.0)	<0.01
CD321/JAM-A	1963.0 (1786.0–2218.0)	1965.0 (1546.0–3121.0)	>0.05
**20% serum**	**controls**	**MI**	**p value**
CD58/LFA-3	1084.5 (959.0–1174.0)	925.0 (580.0–1221.0)	0.02
CD54/ICAM-1	467.0 (443.0–488.0)	450.5 (325.0–805.0)	>0.05
CD321/JAM-A	1965.5 (1634.0–2679.0)	1666.0 (1446.0–2377.0)	<0.01

Data are shown as median (minimum–maximum). Fluorescence intensity [arbitrary units] was measured in cardiomyocytes defined as cells with positive TNNT2 expression. Results are based on n = 13–28 measurements per marker.

**Figure 4:**
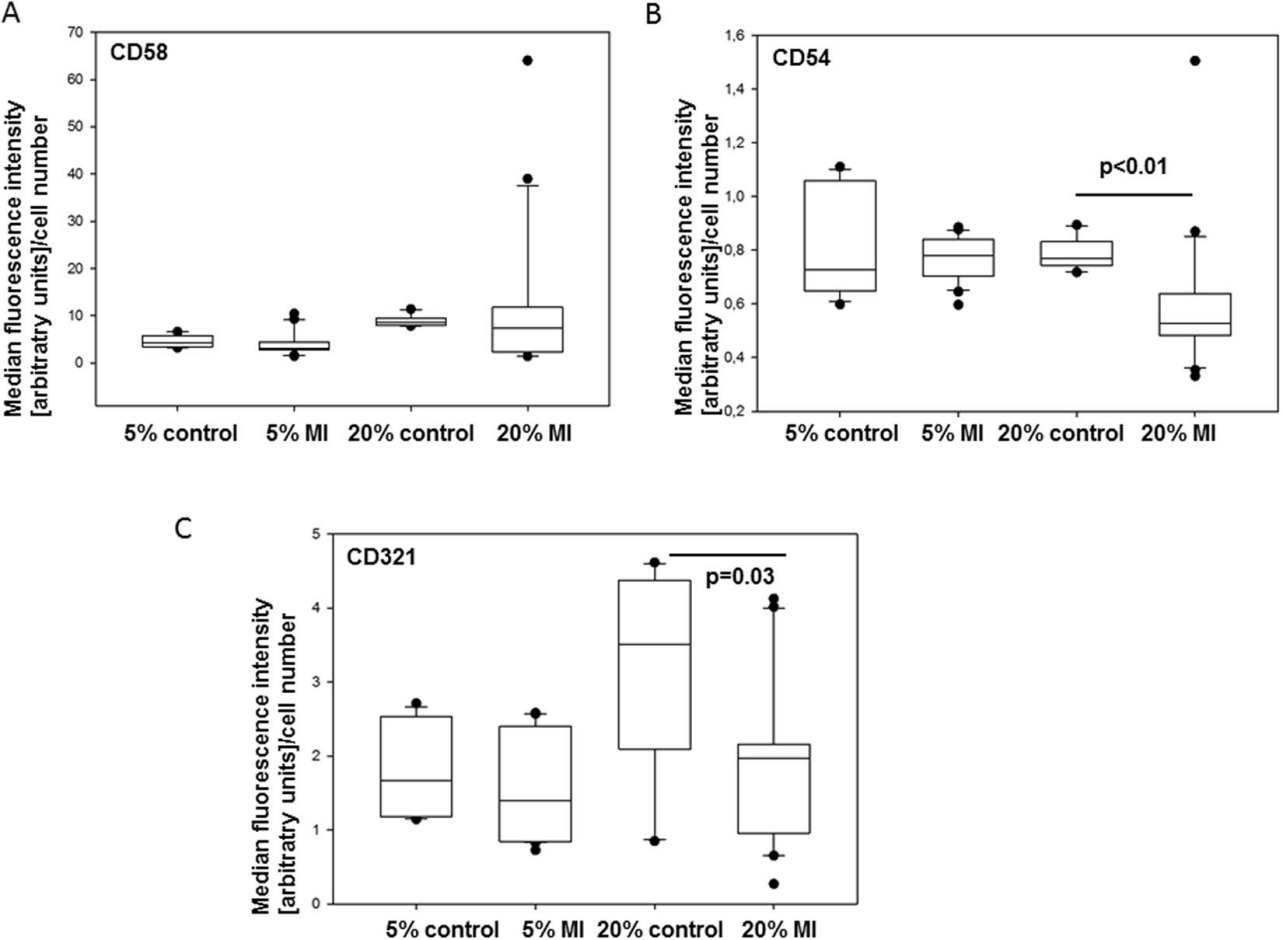
Expression levels (median fluorescence intensity relative to cell number) on iPSC-cardiomyocytes after incubation with myocardial infarction serum (MI) or control serum (5% or 20% v/v). (**A**) CD58/LFA-3, (**B**) CD54/ICAM-1, (**C**) CD321/JAM-A. N = 13–26 measurements per marker. The line within box represents the median, additionally the 10^th^, 25^th^, 75^th^, and 90^th^ percentile is shown; whiskers represent error bars.

To account for changes in cells numbers, the MIF values were normalized to the numbers of cardiomyocytes (defined as cells positive for TNNT2) expressing the respective molecule. CD58/LFA-3 expression levels normalized to cell numbers were not changed by addition to MI serum compared to control serum (p > 0.05, Fig. 4A). In contrast, 20% MI serum reduced per cell-expression of CD54/ICAM-1 when compared to control serum (p < 0.01, Fig. 4B). Similarly, CD321/JAM-A expression per cell was reduced by MI serum of the same concentration (p = 0.03, Fig. 4C).

### Serum of patients with ventricular fibrillation induces higher cell adhesion molecule expression

**Figure 5:**
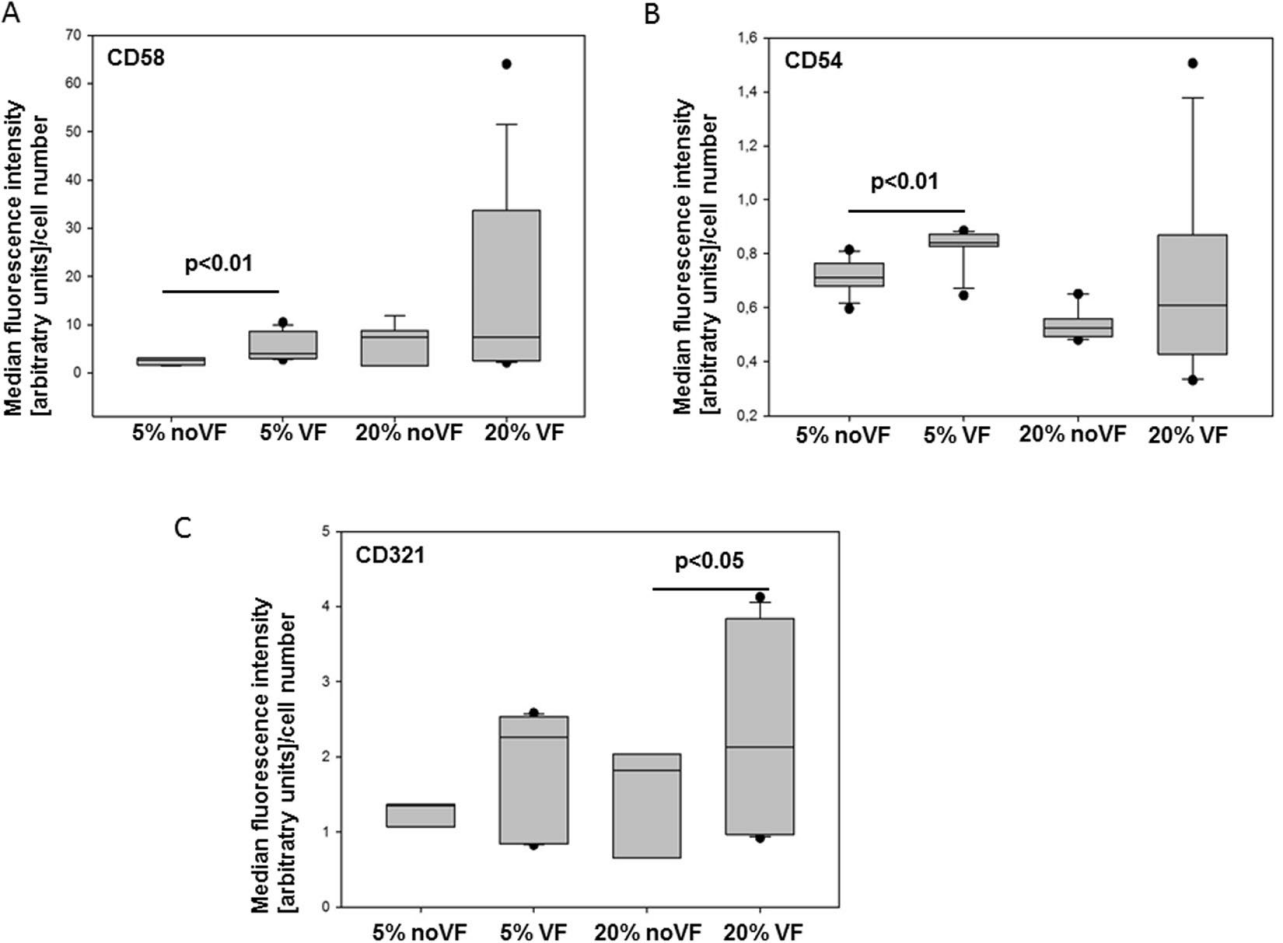
Expression levels (median fluorescence intensity relative to cell number) on iPSC-cardiomyocytes after incubation with 5% or 20% (v/v) myocardial infarction serum with vs. without ventricular fibrillation (VF). (**A**) CD58/LFA-3, (**B**) CD54/ICAM-1, (**C**) CD321/JAM-A. N = 7–15 measurements per marker. The line within box represents the median, additionally the 10^th^, 25^th^, 75^th^, and 90^th^ percentile is shown; whiskers represent error bars.

Ventricular fibrillation (VF) is a complication which can emerge during myocardial infarction. We thus tested whether serum of patients who had developed VF had different effects than serum of patients with myocardial infarction without ventricular fibrillation. VF serum contained higher levels of Il-6 (n = 3; 622 pg/ml [38.4–1339.6] than MI serum without VF (n = 4; 43.2 pg/ml [12.3–71.3]). As expected, if VF serum was used, significantly higher expression of all three cell adhesion molecules was observed compared to serum of patients without VF (Fig. 5).

### Serum of patients with MI does not affect cellular electrophysiology

**Figure 6:**
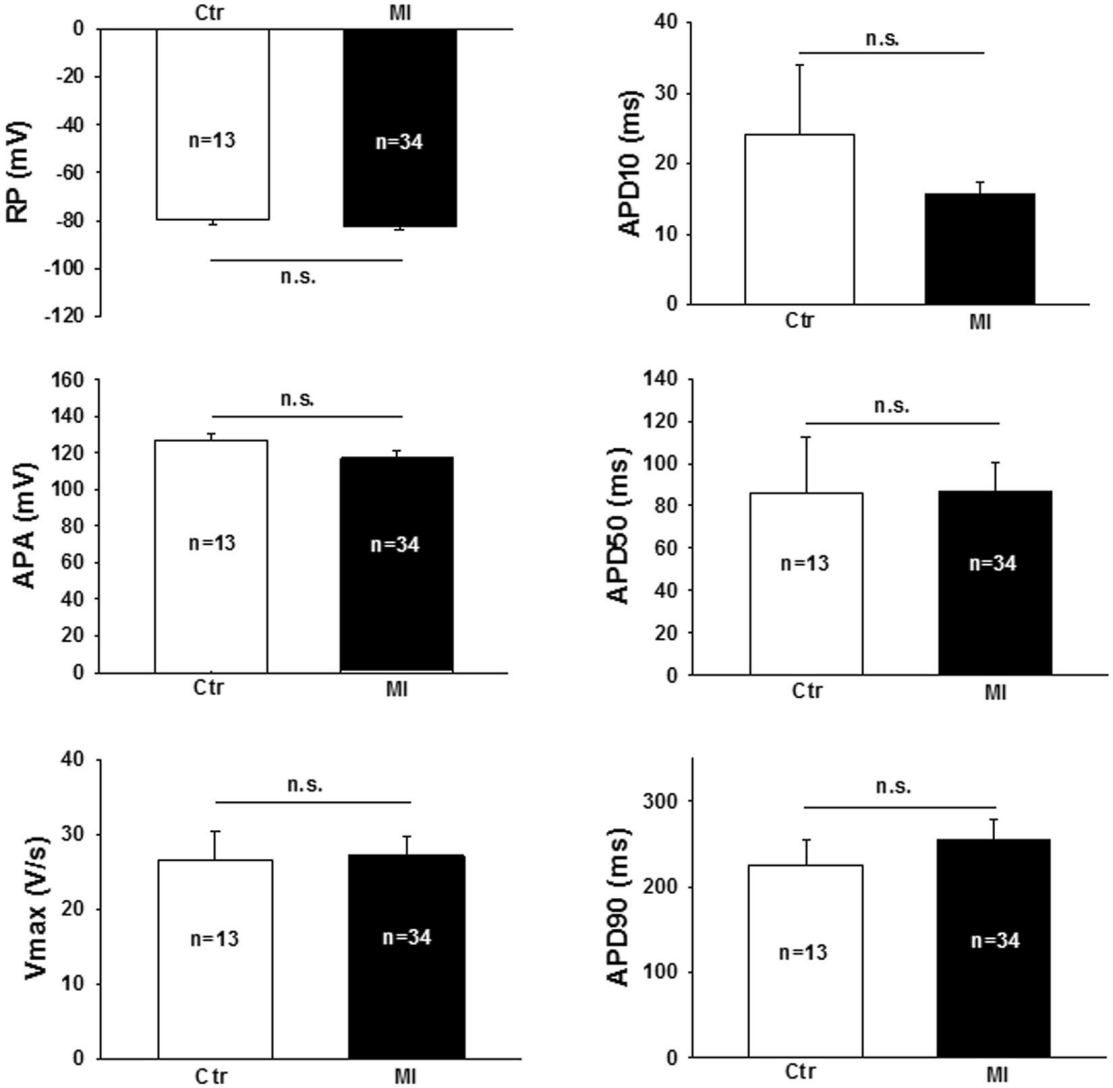
Incubation with myocardial infarction serum (MI, black) does not change electrophysiological properties (control serum – Ctr, white). RP – Resting potential, APA – action potential amplitude, Vmax – maximum depolarization velocity, APD – action potential duration. The mean of the indicated number of measurements is shown; whiskers display standard deviation.

**Figure 7:**
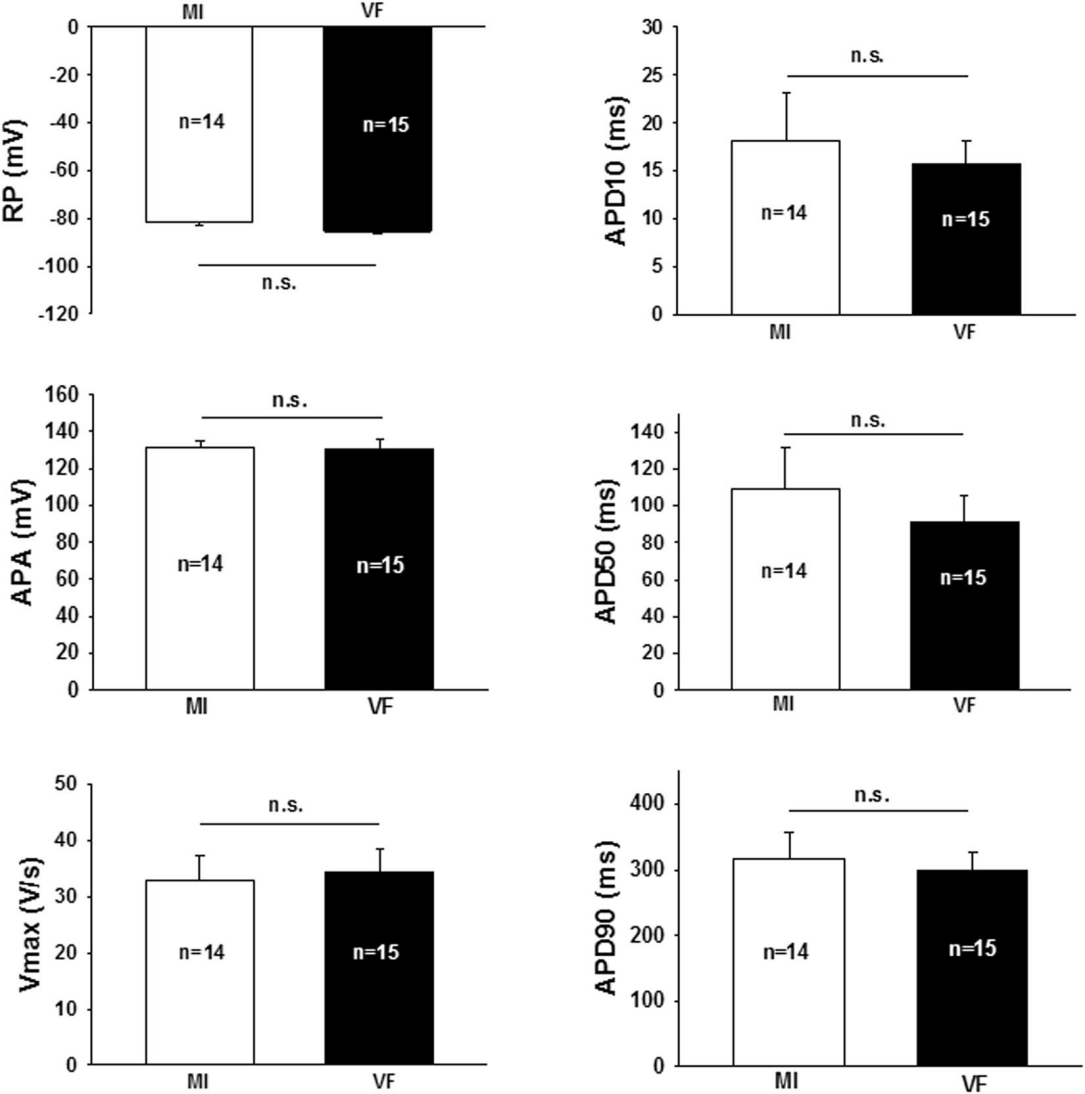
Incubation with myocardial infarction serum with ventricular fibrillation (VF, black) does not change electrophysiological properties (myocardial infarction serum without ventricular fibrillation – MI, white). RP – Resting potential, APA – action potential amplitude, Vmax – maximum depolarization velocity, APD – action potential duration. The mean of the indicated number of measurements is shown; whiskers display standard deviation.

To evaluate the effect of serum on cellular electrophysiology of cardiomyocytes, hiPSC-CMs were incubated with serum of MI patients with or without VF or of controls for 48 hours. Treating cardiomyocytes with either serum had no significant effects on resting potential (RP), action potential amplitude (APA) or duration (APD), or maximum depolarization velocity (Vmax), as shown in Figs 6 and 7.

## Discussion

In the current study, we found for the first time the stable expression of the cell adhesion molecules CD54/ICAM-1, CD58/LFA-3 and CD321/JAM-A on iPSC-cardiomyocytes, and a reduction of the expression levels of these cell adhesion molecules in hiPSC-CMs induced by serum from patients with myocardial infarction.

For studies on cardiac functions, hiPSC-CMs have important advantages over heterologous expression systems such as Xenopus oocytes, human embryonic kidney (HEK) cells and Chinese Hamster Ovary (CHO) cells, which lack important constituents of cardiac ion channel macromolecular complexes that might be necessary for normal electrophysiological characteristics. Animals possess cardiac electrophysiological properties crucially different from that in humans, rendering animal cardiomyocytes not ideal for studies on human physiology and diseases. Therefore, hiPSC-CMs are a good alternative for modeling cardiac diseases and studying drug effects or mechanisms^8^.

Acute myocardial infarction stimulates a massive inflammatory response. The necrotic tissue releases proteins summarized by the term danger-associated molecular patterns (DAMP), which bind to Toll-like receptors and start the inflammatory reaction^3,9^. Chemokines and cytokines are secreted and start invasion of the infarcted area by neutrophils, monocytes and lymphocytes^9^. In the past, several animal studies have shown that non-infarcted myocardium, contrary to the infarcted tissue, does not express pro-inflammatory cytokines^10–12^. One reason might be the fact that in these models the perfusion of the non-infarcted myocardium is intact. However, it is unclear so far how healthy myocardium responds to the pro-inflammatory environment presenting after myocardial infarction. For sepsis, spreading of inflammation and cell damage by contact with septic serum has been shown recently in an *ex-vivo* model^6^, and myocardial cells showed dysfunction after incubation with plasma of patients in septic shock^13^. Interestingly, Youker *et al*. were able to transfer myocardial infarction-associated inflammation to isolated canine cardiomyocytes by incubating the cells with lymph collected directly from the area of ischemic canine myocardial tissue, thereby stimulating ICAM-1 expression as monitored by neutrophil adhesion, an observation attributed to Il-6 content of the lymph as it was deleted by Il-6-antibody^14^. Differences of this study to ours might be caused by the fact that Youker *et al*. used lymph fluid drained directly from the inflamed myocardium which might contain higher levels of pro-inflammatory mediators than serum does, and which might contain especially pro-inflammatory lipoid structures. In contrast, our model of using serum is more comparable to humans with myocardial infarction.

We decided to study three different adhesion molecules having different functions during inflammatory response. By immunostaining, we demonstrated the localization of the molecules in question in the plasma membrane of cardiomyocytes, as proven by co-staining for troponin T^15^. LFA-3 (CD58) is a ligand of the T lymphocyte CD2 protein and mediates adhesion and activation of T lymphocytes. The protein is associated with autoimmune diseases such as multiple sclerosis^16^, and with tumor growth^17^. CD58/LFA-3 was found on intercalating discs of the myocardial syncytium^18^. However, its precise function within the myocardium is not known yet. For CD54/ICAM-1 many studies exist which have underlined the importance of this cytokine for the function of immune response. The primary function of this cytokine is the mediation of neutrophil adhesion to endothelium, rolling and diapedesis^19^. In the heart, CD54/ICAM-1 is released by cardiomyocytes under different conditions of cellular stress, such as hyperoxia^20^, ischemia/reperfusion^21^, hyperglycemia^22^, or viral inflammation^23^. Recently, a positive correlation was noted between expression levels of troponin and CD54/ICAM-1 in biopsies taken from human ischemic myocardium^24^. The expression of CD54/ICAM-1 in ischemic myocardial tissue follows the secretion of DAMP proteins^3^. CD321/JAM-A is an endothelial junction protein which mediates leukocyte diapedesis in ischemic tissues^25,26^. Interestingly, we found that the protein is expressed in 69.6% of cardiomyocytes at day 25 of differentiation. Previously, secretion of the peptide by cardiac progenitor cells was shown^27^. As our cell culture contains a mixture of cells, a “transfer” from other cell types such as endothelial cells cannot be ruled out. However, cardiomyocytes were defined as TNNT2-positive cells in the flow cytometry analysis and immunostaining. To our knowledge, this is the first time that its expression has been described in this cell type.

Interestingly, in our study serum of patients with myocardial infarction affected both the expression levels of the cell adhesion molecules and the number of positive cardiomyocytes. The reduction of expression levels is surprising, as myocardial infarction serum is supposed to contain different pro-inflammatory mediators. Indeed, Il-6 was elevated in samples of patients with myocardial infarction compared to control samples. Thus, healthy cardiomyocytes do not react by changing to an inflammatory phenotype upon stimulation with pro-inflammatory serum, but rather suppress inflammation. Recently, we showed that several cell adhesion molecules were induced by incubating iPSC-derived cardiomyocytes of healthy donors with lipopolysaccharides^7^. Obviously, pathways are present in iPSC-cardiomyocytes which are specialized to cell responses depending on stimulus.

Even more interesting is the finding of an effect of myocardial infarction serum on the numbers of cell numbers expressing the different cell adhesion molecules. While some cells downregulated the respective pathways after incubation with myocardial infarction serum, the response of CD58/LFA-3 was completely abolished in up to 50% of cells. When translated to myocardial tissue placed in a heart subjected to infarction at a remote area, this would imply a reduction of the “appeal” of healthy myocardium to T lymphocytes, especially in contrast to the highly “attractive” infarcted area. In accord, the stimulation of neutrophils takes place on a low level in healthy myocardium, as the CD54/ICAM-1 expression per cell was reduced by MI serum.

Ventricular fibrillation (VF) is a complication of myocardial infarction occurring in up to 12% of cases^28^, although many cases of sudden cardiac death might be based primarily on death due to arrhythmia, giving the assumption of even higher a number. The genesis of VF during myocardial infarction is probably multifactorial, as local necrosis^29^, cardiac sympathetic nerve stimulation^30,31^ and also several clinical and demographic pre-disposing factors, such as alcohol intake or pre-existing atrial fibrillation^32^, are discussed. Recently, an association of previous episodes of ventricular tachyarrhythmia and elevated markers of inflammation was described in patients with stable coronary artery disease^33^. In an animal model, ischemia-triggered VF was associated with higher plasma levels of TNF-α than electrically induced VF, ascribed to the ischemia of the myocardium and the prolonged resuscitation phase^34^. In our study, serum of patients with VF was less able to reduce cell adhesion molecule expression. Indeed, VF serum contained higher levels of Il-6 than MI serum without VF, although only small numbers are available from our patients group. As we do not have the blood levels of Il-6 prior to the event, it is impossible to discern whether this serum property is a cause or a consequence of VF during myocardial infarction. Whether there is a serum component pre-disposing to VF after myocardial infarction or whether the VF event changes the serum towards a more inflammatory phenotype remains to be clarified.

Based on our results, one can speculate that in “healthy parts” of the myocardial tissue pro-inflammatory signals are actively suppressed to protect against spreading of the tissue inflammation. Serum of patients with VF seems to be more pro-inflammatory, translating into a higher risk of inadequate infarct containment for the affected myocardium. Although adhesion molecule expression was affected by serum of myocardial infarction, the electrophysiological properties of the cardiomyocytes involved in the generation of arrhythmias were not influenced by serum incubation. This is interesting, as several soluble mediators accumulating in ischemic conditions participate in the generation of arrhythmias occurring during reperfusion injury^35,36^. Although we determined only interleukin-6 levels in our samples, one can assume based on the blood chemistry values of the patients that the MI samples consisted the “typical” molecules found after reperfusion. Our results clearly show that serum of MI patients has no effects on action potentials and thus probably no proarrhythmic effect on healthy cardiomyocytes. The reason for the arrhythmogenesis after MI needs to be further investigated. Of further interest is the reaction of cardiomyocytes generated from stem cells of patients carrying a structural alteration, such as hypertrophic or dilated cardiomyopathy or arrhythmogenic rightventricular cardiomyopathy. In these cells, adhesion molecule expression and response might be different from healthy cardiomyocytes. The study of these cells is currently under way in our laboratory. Further studies are also needed to elucidate the signal transduction of inflammation relating to the cell adhesion molecules in cardiomyocytes.

### Summary

Myocardial infarction serum exerts anti-inflammatory effects on healthy iPSC-cardiomyocytes, while serum of patients with myocardial infarction and VF is less effective. MI serum or VF serum does not change electrophysiological properties of the cells. Further studies of the innate immunofunction of cardiomyocytes are needed to define the underlying pathways.

### Limitations

The blood samples were gathered after restoration of flow, thus, after reperfusion, not during the ischemic phase of the myocardial infarction. In a strict sense, the results might therefore represent the effects of an ischemia-reperfusion injury rather than “pure” myocardial ischemia. However, due to the necessity of immediate treatment, sample collection had to be postponed until stabilization of the patients. We tested the effect on healthy cells living in a mixed cell culture setting. This is of course a different setting than cardiomyocytes contained in myocardium of an individual surviving an ischemic event of a distant area. We measured whole-cell-expression of the adhesion molecules, not only surface expression, which does not represent the presentation of the molecules to cells of the immune system.

## Materials and Methods

### Ethics statement

The skin biopsies from the healthy donors were taken after written informed consent had been obtained. The study was approved by the Ethics Committee of the Medical Faculty Mannheim, University of Heidelberg (approval number: 2009–350N-MA) and by the Ethics Committee of University Medical Center Göttingen (approval number: 10/9/15), and carried out in accordance with the approved guidelines.

### Serum generation

Venous blood of patients presenting with acute myocardial infarction (ST-segment elevation infarction, STEMI, or non-ST-segment elevation infarction, NSTEMI) was collected within the first 12 hours after the event in serum monovettes® and centrifuged. The serum was stored at −80 °C until further use. Myocardial infarction (STEMI or NSTEMI) were defined according to the guidelines of the European Society of Cardiology^37,38^. For control, serum of three healthy volunteers was collected using the identical centrifugation protocol. All volunteers presented no clinical manifestation of CAD or of atherosclerosis at other sites, and were taking no medication. The study was approved by the Ethics Committee of University Medical Centre Mannheim and was conducted in accord with the Declaration of Helsinki.

### Enzyme-Linked Immunosorbent Assay

Interleukin-6 (Il-6) was measured according to manufacturer’s instructions in serum of patients and controls with an enzyme-linked immunosorbent assay (ELISA) kit (RayBio, USA). All measurements were done in duplicate.

### Human iPS cells

Human iPS cells (hiPSCs) were generated from primary human fibroblasts derived from skin biopsies of two different healthy donors. For hiPSC line Donor 1 (D1), the reprogramming factors OCT4, SOX2, KLF4 and c-MYC were transfected using lentivirus particles carrying the transactivator rtTA and an inducible polycistronic cassette, as previously described^39,40^. Donor 2 (D2)-iPSC were generated using the episomal 4-in-1 CoMiP reprogramming plasmid (OCT4, KLF4, SOX2, c-MYC and short hairpin RNA against p53) to reprogram the primary cells into iPSCs in feeder free culture conditions.

### Generation of hiPSC-cardiomyocytes

HiPSCs were cultured under feeder free conditions and differentiated into iPSC-cardiomyocytes as described previously^8^. In brief, culture flasks and dishes were coated with Matrigel (Corning). hiPSCs were cultured in TeSR-E8 media (Stemcell Technologies) and changed to RPMI 1640-Glutamax (Life Technologies) containing sodium pyruvate, penicillin/streptomycin, B27 (Life Technologies) and ascorbic acid (Sigma Aldrich) after start of the differentiation process. Differentiation into CMs was induced by addition of CHIR99021 (Stemgent), BMP-4 (R&D Systems), Activin A (R&D Systems), FGF-2 (Miltenyi Biotec) and IWP-4 (Stemgent) at different time points to the medium. Selection of cardiomyocytes occurred during the third week of cultivation by changing the medium to a lactate-supplemented (Sigma, Germany) RPMI-medium free of glucose and glutamine (WKS, Germany). Afterwards, cells were fed with RPMI 1640-Glutamax containing sodium pyruvate, penicillin/streptomycin, B27 and ascorbic acid until termination of the experiments.

### RNA extraction, cDNA synthesis and quantitative real-time PCR

RNA was extracted from hiPSC-CMs by lysis with RLT lysis buffer, followed by the application of the RNeasy MiniKit (Qiagen) according to manufacturer’s instructions. cDNA synthesis was performed with oligo (dT) primers using AMV reverse transcriptase (Roche). For quantitative real time PCR, hot start Taq DNA-polymerase and SYBR-Green were used, together with commercially available primers (GAPDH, #PPH00150F; TNNT2, #PPH025619A; POUF51, #PPH02394E, Qiagen). The mean CT value of 3 to 6 biological replicates of three different differentiations was calculated from three technical replicates. Normalized mRNA expression was calculated by using ΔCT = (CT_gene of interst_ − CT_housekeeping gene_).

### Incubation studies and flow cytometry analysis

Cardiomyocytes at differentiation day 25 and older were incubated with cell medium supplemented with 5% or 20% (v/v) of serum of patients or controls for 48 hours at 37 °C, 5% CO_2_^41^. Cells were detached from wells by incubation with collagenase I (CLS 1, Worthington, Cat Nr. LS004196, 250 U/mg) for 40 minutes at 37 °C. Cells were washed twice with PBS and 0.05% trypsin. Afterwards, cells were fixated in 4% formaldehyde (10 minutes, 20 °C) and permeabilized (Perm/Wash Buffer, BD). After another wash, antibodies were added and incubated for 30 minutes at 4 °C in the dark (BD Pharmingen: anti-CD62-PE,# 555524, anti-CD58-FITC #555920, anti-CD321-PE, #552556; Antibodies online: anti-CD102-FITC, #ABIN1383720). After washing, measurements of fluorescence were performed on a FACS Canto II, BD. Analysis of measurements was performed with the software FlowJo 10.1. Flow cytometry measurements were done by a technician blinded for the study protocol. Expression was defined “positive” if more than 2% of cardiomyocytes were stained with the respective marker.

### Immunofluorescence staining

hiPSC-CMs were grown on culture slides (Falcon) and allowed to rest for at least 2 days. Cells were fixed with 4% formaldehyde and permeabilized with 0.5% triton-X, followed by incubation with antibodies against cell adhesion molecules (anti-CD54-FITC, ABIN #2144636; anti-CD58-FITC, BD #555920; anti-CD321-PE, BD #552556, 1 hour, room temperature). Afterwards, staining of cardiac troponin T was performed with an AlexaFluor647-conjugated antibody (BD #565744, 1 hour, room temperature), followed by nuclear staining with DAPI (Vector, #H-1200). Photographs were taken with a Leica DMRE microscope (Leica Application Suite V4.4.0, Microsystems CMS GmbH, Switzerland).

### Patch clamp

Action potential characteristics were measured by standard patch-clamp recording techniques in the whole-cell configuration at room temperature according to a recently published protocol^42^. For measurements, 3 randomly chosen serum samples of each group were taken, and measurements were performed in at least 13 single hiPSC-CMs per group.

### Statistics

Data are presented as number (percent) for ordinal data or mean + −SD or as median (minimum-maximum) for continuous data, depending on data distribution. Group comparison was done with student’s t-test or Mann-Whitney-U-rank sum test. Multiple comparisons were done with ANOVA on ranks. P values are understood to be strictly descriptive. Statistical significance was assumed for p < 0.05. All analyses were done with SigmaPlot Version 13.0.

### Ethics approval and consent to participate

The study was approved by the Ethics Committee of the Medical Faculty Mannheim, University of Heidelberg (approval number: 2009-350N-MA) and by the Ethics Committee of University Medical Center Göttingen (approval number: 10/9/15), and carried out in accordance with the approved guidelines. Written informed consent was obtained from all participants or their legal representatives.

## Data Availability

The datasets used and/or analyzed during the current study are available from the corresponding author on reasonable request.
